# Chitosan Application in Vineyards (*Vitis vinifera* L. cv. Tinto Cão) Induces Accumulation of Anthocyanins and Other Phenolics in Berries, Mediated by Modifications in the Transcription of Secondary Metabolism Genes

**DOI:** 10.3390/ijms21010306

**Published:** 2020-01-02

**Authors:** Rupesh Kumar Singh, Viviana Martins, Bruno Soares, Isaura Castro, Virgílio Falco

**Affiliations:** 1 Centro de Química de Vila Real (CQ-VR), Universidade de Trás-os-Montes e Alto Douro (UTAD), 5000-801 Vila Real, Portugal; vfalco@utad.pt; 2 Departamento de Agronomia, Universidade de Trás-os-Montes e Alto Douro (UTAD), Quinta de Prados, 5000-801 Vila Real, Portugal; bmgoncalves90@gmail.com; 3 Centro de Biologia Molecular e Ambiental (CBMA), Department of Biology, UMINHO, 4710-057 Braga, Portugal; vvymartins@gmail.com; 4 Centro de Investigação e Tecnologias Agro-Ambientais e Biológicas (CITAB), Universidade de Trás-os-Montes e Alto Douro (UTAD), Quinta de Prados, 5000-801 Vila Real, Portugal; icastro@utad.pt; 5 CoLAB Vines&Wines, Associação para o Desenvolvimento da Viticultura Duriense (ADVID), Régia Douro Park, 5000-033 Vila Real, Portugal

**Keywords:** chitosan, elicitor, *Vitis vinifera* L., anthocyanins, polyphenols, tannins, transporter genes

## Abstract

Despite the numerous beneficial properties and uses of chitosan in agriculture, the molecular mechanisms behind its elicitation potential are still unclear. This study aimed at understanding the effect of chitosan application in the levels of phenolic compounds of *Vitis vinifera* L. red grapes berry skin (cv. Tinto Cão) during veraison. Grapevines were treated with chitosan (0.01% in 0.01% acetic acid) while control grapevines were sprayed with 0.01% acetic acid. Results showed that several monomeric anthocyanins increased significantly in berry skins after treatment with chitosan. Additionally, Catechin, Rutin and Querecetin-3-*O*-galactoside were also recorded in higher amount upon chitosan treatment. Besides modulating the phenolic content, chitosan treatment also induced modifications in several target genes encoding key enzymes and transporters involved in secondary metabolic pathways. For instance, the genes *PAL*, *CHS*, *F3H*, *ANR*, *UFGT*, *ABCC1*, *GST*, *MATE1* were upregulated in leaves and berry skins at veraison cessation in response to chitosan treatment. Overall, the results demonstrated that chitosan has a stimulatory effect on the accumulation of phenolic compounds, including anthocyanins, mediated by modifications in the transcription of key genes involved in their biosynthesis and transport in grape berries.

## 1. Introduction

Owing to multifarious properties, chitosan has become one of the most popular biopolymer and leading elicitor molecule for plants since the last decade [1,2]. It is produced by alkaline deacetylation of chitin obtained from insects, exoskeleton of crustaceans, and fungal cell walls [3]. In other words, the raw material for chitosan production is abundant in nature, and is second only to cellulose in terms of the availability. Hence, chitosan application is economically feasible for various applications in agriculture [1]. The study conducted by Allan and Hadwiger [4] reported the ability of fungal cell wall components to induce a plant’s inherent immune system which ultimately enhances pathogen prevention. This work was considered as a pioneer study and opened new frontiers for the application of fungal cell wall derivatives, such as chitosan, to achieve desired results in agriculture.

The potential of chitosan application has been studied in many horticultural crops, towards inducement of defense mechanisms, improved agronomic traits, easier post-harvest management of fruits and vegetables, augmented plant growth and physiological activities, including as a bio-fertilizer/fertilizer protectant [5], and enhanced abiotic stress management properties [6]. Some reports demonstrated its potential effect on powdery mildew [7] and gray mold control [8] in grapevine. For instance, increased PAL activity has been reported in chitosan-treated grape leaves against *Botrytis cinerea* [9]. Previous studies also demonstrated that continuous chitosan application improved polyphenolic content and wine antioxidant potential when compared with the traditional fungicide treatments [7], whereas preharvest application showed no differences in these parameters [10]. Our recent study further suggested improved antioxidant potential upon chitosan application in *Vitis vinifera* L. [11].

Grape phenolic composition and fruit development are highly modulated during the veraison stage, especially in terms of anthocyanins, proanthocyanidins and flavonols content [12,13], and studies comprising the effect of chitosan application in grapevines during this stage are scarce, particularly at the molecular and biochemical level. Thus, the present study investigated the effect of chitosan application (0.01% in 0.01% acetic acid) at the beginning of veraison on the levels of phenolics including anthocyanins in *Vitis vinifera* L. cv. Tinto Cão. Control grapevines were treated with 0.01% acetic acid solution to ensure the invigorated effect of chitosan; which was left behind in our previous study [11]. The study was complemented with expression studies of target genes encoding key enzymes of secondary metabolite synthesis and transport in grape berry skins, including phenylalanine ammonia lyase (*PAL*) that catalyzes the first step of phenylpropanoid biosynthesis, *UFGT* that mediates the limiting step towards anthocyanin biosynthesis and *MATE1* that transports anthocyanins to the vacuole. In addition, transcript levels of genes encoding antioxidant enzymes were also studied, including *Cu/Zn-SOD* and *Fe-SO*D.

The present study validated the application timing for chitosan application towards grape berry qualitative improvement in the field, and further elucidated the molecular mechanisms of chitosan action in the secondary metabolism in the grapevine.

## 2. Results and Discussion

**Figure 1:**
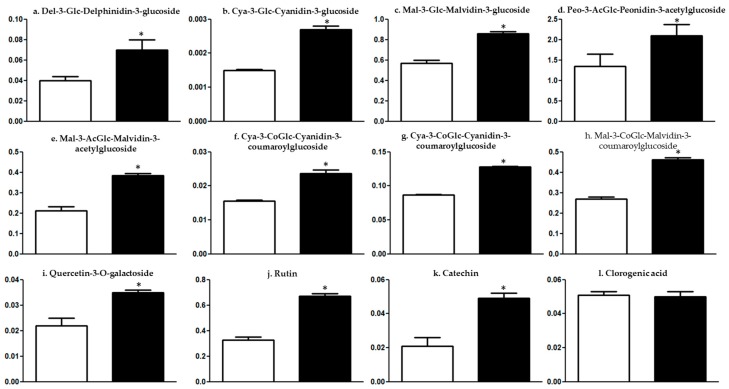
Effect of chitosan application at veraison cessation stage on different monomeric anthocyanins (µg/100 mg dry wt.) and other phenolic compounds (µg/100 mg dry wt.) in *Vitis vinifera* L. cv. Tinto Cão grape berry skins, where white column is always control sample and black column is always treatment (**a**–**l**). Treated samples represent the chitosan applied berry skins (aqueous solution of 0.01% chitosan in 0.01% acetic acid) and control samples represent 0.01% aqueous acetic acid application. Results show mean ± SD from three biological replicates and asterisks represent the significance level as compared to control, by the Students *t*-test: * *p* < 0.05.

Elicitors are the molecules which stimulate the synthesis of secondary metabolites in plants following their applications [14]. Chitosan was used as an elicitor in the present study and different monomeric anthocyanins and some other phenolic compounds were measured in the berry skins. Delphinidin-3-glucoside was recorded as 75% higher (Figure 1a), 80.02% in Cyanidin-3-glucoside (Figure 1b), 50.87% in Malvidin-3-glucoside (Figure 1c), 56.72% in Peonidin-3-acetylglucoside (Figure 1d), 82.45% in Malvidin-3-acetylglucoside (Figure 1e), 51.92% in Cyanidin-3-coumaroylglucoside (Figure 1f), 47% in Peonidin-3-coumaroylglucoside (Figure 1g) and 71.74% increase in Malvidin-3- coumaroylglucoside (Figure 1h) was observed in treated berry skins in comparison to control. Additionally, Querecetin-2-*O*-galactoside was recorded as 59.09% higher (Figure 1i), Rutin as 105.85% (Figure 1j) and 133.33% increased Catechin (Figure 1k) in chitosan-treated berry skins in comparison to control, although Chlorogenic acid did not gave any significant difference (Figure 1l).

Veraison is a crucial phenotypic stage and a tipping point in red grapevines when grape berries start gaining color due to anthocyanins, and other phenolic compounds also start accumulating in berry skins. Increased synthesis of anthocyanins and other phenolics in berries, following the application of chitosan, highlights its potential of eliciting the secondary metabolites’ pathways during veraison.

Results are in accordance to previous studies that showed the stilbenes and anthocyanin content increasingly accumulated upon chitosan application (0.1% acidic solution) in *V. vinifera* Barbera grape cell suspension cultures [15]. In contrast, foliar application of 0.03% acidic chitosan solution in *V. vinifera* ‘Tempranillo’ grapes did not have a significant increase in the phenolics and anthocyanin contents [16]. The present study strengthens previous findings and validates that 0.01% chitosan application promotes secondary metabolism, highlighting that elicitor concentration and timing of application are very important in order to get optimum effects. Our previous study [11] has shown improved metabolites accumulation as well as antioxidant potential in different phenotypic stages where control grapevines were maintained without any acetic acid spray. The present study incorporated a similar amount of acetic acid (0.01% acetic acid in water) solution application in control grapevines, followed by identification of different monomeric anthocyanins as well as rutin, catechin, chlorogenic acid and quercetin-3-*O*-galactoside. All the identified compounds were recorded in increased concentration in chitosan-treated grapevines except chlorogenic acid. Thus, present findings confirm the elicitation potential of chitosan and rule out the possible effect of acetic acid in chitosan solution.

To investigate the molecular mechanisms underlying the increased content in phenolic compounds including anthocyanins, the expression of target genes involved in secondary metabolism pathways were studied. The following enzymes were selected due to their involvement in major metabolic steps: phenylalanine ammonia lyase-*PAL*, chalcone synthase-*CHS*, flavanone 3-hydroxylase-*F3H*, anthocyanidin reductase-*ANR*, UDP-glucose: flavonol 3-*O*-glucosyl transferase–*UFGT*, anthocyanin transporters *ABCC1* and *MATE1*, and glutathione *S*-transferase-*GST* involved in anthocyanin uptake to the vacuole. The gene expression was evaluated in berry skins as well as in leaves.

**Figure 2:**
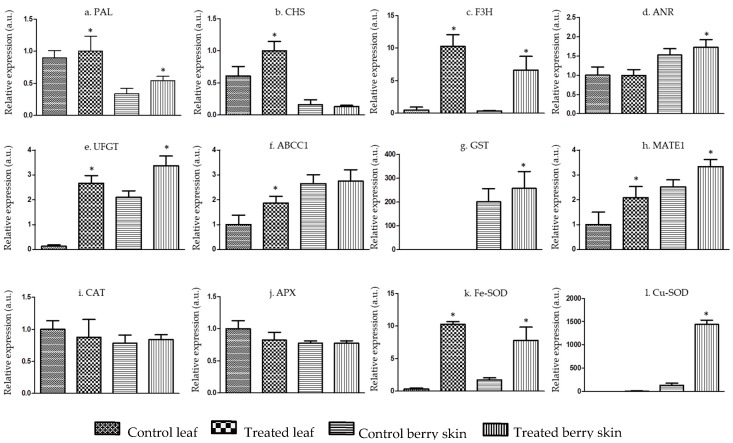
Effect of chitosan on the expression of target genes encoding enzymes of secondary metabolism, anthocyanin transport and antioxidant status, in *Vitis vinifera* L. cv. Tinto Cão leaves and berry skins (**a**–**l**): phenylalanine ammonia lyase-*PAL*, chalcone synthase-*CHS*, flavanone 3-hydroxylase-*F3H*, anthocyanidin reductase-*ANR*, UDP-glucose: flavonol 3-*O*-glucosyl transferase–*UFGT*, anthocyanin transporters *ABCC1* and *MATE1*, glutathione S-transferase-*GST*, catalase-*CAT*, ascorbate peroxidase-*APX*, superoxide dismuatase-*Cu/Zn-SOD* and *Fe-SOD*. Treated samples represent the chitosan applied leaves and berry skins (aqueous solution of 0.01% chitosan in 0.01% acetic acid) and control samples represent 0.01% aqueous acetic acid application. Results show mean ± SD from three biological replicates and asterisks represent the significance level as compared to control, by the Students *t*-test: * *p* < 0.05.

*PAL* enzyme catalyzes the first step in the phenylpropanoid pathway initiating phenolic biosynthesis [17]. In the present study, the expression of *PAL* increased by 0.6-fold in grape berry skin and by 0.2-fold in leaves upon chitosan application (Figure 2a). Zhao et al. [18] reported that *PAL* expression increased from veraison and reached to the maximum at maturity, suggesting its positive role in anthocyanin accumulation. Accordingly, fruit color development and *PAL* expression correlated positively in earlier reports [19,20], while Chen et al. [21] reported a sharp increase until maturation and a constant decrease thereafter.

Chalcone synthase (*CHS*) is a key enzyme for flavonoid biosynthesis in *V. vinifera* red grapes, and gene expression generally increases after veraison until maturity in grape berries [19,22]. In the present study, an increase of 0.6-fold in grapevine leaves was observed upon treatment with chitosan, while no significant differences were observed in grape berry skins (Figure 2b). Although previous studies showed a positive correlation between *CHS* expression and anthocyanin synthesis in grape berries at maturity [18]), results in the present study showed no direct relationship between *CHS* expression and anthocyanin content in berries upon treatment with chitosan. Nonetheless, a close relationship was observed between the expression of *CHS* and the levels of total phenolics and tannins, suggesting that it is mediating the synthesis of these metabolites in leaves.

Flavanone 3-hydroxylase (*F3H*) was reported to be differentially expressed throughout grape berry fruit development, and sharp upregulation from veraison till maturity [23]. The present study recorded a drastic change of *F3H* transcript accumulation in leaves (23.34-fold increase) and 19.16- fold increase in grape berry skins in treatment of grapevines with chitosan in comparison to control (Figure 2c). Another study revealed its differential expression before starting of veraison and an upregulation later till complete maturity, and correlated it directly for flavanone synthesis in grapevines [18]. The present study recoded a drastic shift in transcript accumulation of *F3H* in leaves as well as grape berries upon chitosan application, and suggests that chitosan induces the flavone synthesis at the molecular level in grapevines.

Anthocyanin reductase gene (*ANR*) was also investigated and the differential expression was observed non-significant upon chitosan treatment (Figure 2d). UDP-glucose: flavonol 3-*O*-glucosyl transferase (*UFGT*) is a key factor in anthocyanin biosynthesis in red grapes and has been reported in grape skins only after veraison [19,20,24]. The present study demonstrates very low expression of UFGT transcript in control leaves whereas a huge shift (22.5-fold increase) in treated leaves. A 1.6-fold increase in grape berry skin in comparison to control was also noticed (Figure 2e). Zhao et al. [18] reported upregulation of this key enzyme from veraison to maturity in grape berries, thus establishing a positive correlation of this gene in anthocyanin biosynthesis, especially monoglucosides. The present study showed drastic change in expression of this gene in leaves as well as significant increase in grape berry skins and evidenced the invigorated effect of chitosan application towards induced anthocyanin biosynthesis pathway.

The three key transporter genes were investigated and *ABCC1* expression doubled in leaves, while there was no significant change in berries upon chitosan application (Figure 2f). Expression of another transporter (*MATE1*) was doubled in leaves, whereas it increased 0.8-fold in berry skins in treated vines (Figure 2h). Glutathione *S*-transferase (*GST*) specifically expressed only in berry skins, observed a 0.8-fold increase in treated vines, while no expression in leaf tissues (Figure 2g). Anthocyanins are specialized metabolites in *V. vinifera* berries and tend to accumulate in cellular vacuole by vacuolar sequestration, an evolved strategy by higher plants [25]. This mechanism helps in overcoming the negative effects of high cytosolic accumulation and feedback inhibition of the end product in the cell [26]. The tonoplast transporters (*ABCC1* and *MATE1*), and *GST* were observed increasingly upregulated in grape berries upon kaolin foliar application towards improved anthocyanin accumulation in berry skins [27]. Recently, the roles of *ABCC* and *MATE* transporters in vacuolar accumulation of specific metabolite crocins in saffron stigmas were characterized [28]. Glutathione S-transferase (*GST*) tends to express only in berries and facilitates the conjugation of anthocyanins with reduced glutathione and transports them to the vacuoles [29]. Increased expression of these reporter genes upon chitosan application in the present study suggests a positive elicitation. 

Improved metabolites in plants may lead to increase in antioxidant activity towards higher plant fitness by scavenging the toxic reactive oxygen species [11]. Catalase and Ascorbate peroxidase gene expression was recorded with no significant change in treated plants in comparison to control (Figure 2i,j). Copper/Zinc SOD (Cu/Zn-*SOD*) expression was also measured upon chitosan application and a significant expression increase in leaves (7.6-fold), and a higher accumulation in grape berry skins (11 fold) of treated vines, in comparison to control, was recorded (Figure 2l). *Fe-SOD* also showed an increase in leaves (32-fold) as well as in grape berry skins (4.6-fold) in treated samples in comparison to control (Figure 2k). Superoxide dismutases (*SOD*s) are key players in the plant defense mechanism against reactive oxygen species (ROS), as they catalyze the dismutation reaction [30,31], and have been reported to cope oxidative stress in roots, leaves, seeds, and fruits [32]. In a very recent study, six *Cu/Zn-SODs* and two *Fe-SODs* were identified in grapevine and the expression analysis established a clear correlation of these genes in light stress resistance as well as in different aspects for abiotic stress management [33]. Other recent studies demonstrated a key role of *SOD*s towards different environmental stresses in plants, such as ethylene, salinity, drought and low temperature [34,35] in various monocots and dicots species including *Arabidopsis thaliana* [36], *Dimocarpus longan* [34], *Sorghum bicolor* [37], *Populus trichocarpa* [38], *Musa acuminate* [33], *Gossypium raimondii* and *Gossypium arboreum* [39].

Chitosan is soluble only in an acidic medium and contains traces of solvent material during application; although the amount is very less. One report demonstrated some elicitation potential in Hypericum perforatum root cultures on xanthone biosynthesis [40]. The same amount of acetic acid solution (0.01% aqueous solution) was applied on the control grapevine in the present study to confirm the mechanism of the chitosan effect, which has been unanswered in our previous report [11]. It may be that the difference in xanthone synthesis in cultures may be due to change in culture medium properties by acetic acid and will need further investigation [40].

## 3. Materials and Methods

### 3.1. Plant Material, Treatment and Sample Collection

*Vitis vinifera* L. cv. Tinto Cão was under investigation in present study in the field area. Vineyards were defined in the University of Trás-os-Montes and Alto Douro, (41°19′ N, 7°44′ W, 500 m above mean sea level), Baixo Corgo sub-region of the Demarcated Douro Region, northern Portugal. Three lines that were phenotypically similar were marked (consisting of 12 plants each) for chitosan treatment while similar three lines were marked as control. The vineyard land has morainic, grass-covered, loamy sand, 15% gravel and trained according to the Guyot system.

Fungal originated chitosan was received from Kitozyme (Belgium) and an acidic solution of 0.01% (*w*/*v*) in 0.01% acetic acid was prepared for application. Aqueous 0.01% acetic acid solution was prepared for control grapevines as well. Three marked grapevine lines were sprayed (leaves and berries) with chitosan solution and a similar three lines were sprayed with control solution, at the beginning of veraison (as soon as the berries start coloring). Leaves and berries were collected after complete veraison (all the berries were colored); control samples were collected similarly and frozen immediately in liquid nitrogen. A total of 100 berries were picked randomly in equal proportion and 12 leaves as well for further experiments. Average temperature, humidity, and wind velocity was recorded as 23.4 °C, 57.4%, and 6.5 km/h, respectively, during the application until sample collection. Frozen leaves and berry skins (skins were dissected from berries) from treated and control grapevines were grinded in liquid nitrogen for RNA extraction and one set of frozen berry skins were freeze-dried for extract preparations.

### 3.2. Extract Preparation

Extracts were prepared from 40 mg of freeze-dried grounded berry skin tissue in triplicates. Grounded tissue was mixed with 950 µL of 70% methanol and 50 µL of internal standard solution (naringin, Sigma-Aldrich, Tauferkichen, Germany), followed by incubation in a water bath at 70 °C for 30 min with constant stirring. Extracts were centrifuged at 11,000 rpm for 20 min at 4 °C (Centrifuge 5804R, Eppendorf, Hamburg, Germany) and supernatants were filtered through PTFE 0.2 µm, Ø 13 mm (Teknokroma, Barcelona, Spain) taken out in separate glass HPLC vials. Extracts were stored in −20 °C until further analyses.

### 3.3. HPLC-DAD-UV/VIS Analysis

The method was adopted from Aires et al. with slight modifications [41]. In brief, a HPLC (Gilson) system equipped with one mixture chamber (Gilson, model 811A), two pumps (Gilson, model 305 and 306), automatic injector (Gilson, model 231X), oven (Jones chromatography) and a diode array detector (DAD) (Thermo, Finnigan Surveyor detector) was used to identify the polyphenols present in grape berry skins. The mobile phase was composed of water with 0.1% of trifluoroacetic acid (TFA, solvent A) and acetonitrile with 0.1% TFA (solvent B). 10 µL of each extract was injected into a C18 column (250 × 4.6 mm, 5 μm particle size, ACE, Advanced Chromatography Technologies, Aberdeen, UK). The elution was performed at a flow rate of 1 ml/min with gradient: 0 min 100% A, 5 min 100% A, 15 min 80% A, 30 min 50% A, 45 min 0% A, 50 min 0% A, 55 min 100% A, and 60 min 100% A. Catechin, chlorogenic acid, rutin/querecetin and anthocyanins were detected at 280, 320, 370 and 520 nm, respectively. Compounds were identified through peak retention time, UV spectra and UV maxima absorbance bands, as well as by comparison with commercial external standards. Quantification was done using commercial standards of the identified compounds. The results were expressed as µg/100 mg dry wt. All chemicals used in this study were of analytical grade (Thermo Fisher, Porto, Portugal) and standards were purchased from Extrasynthèse (Genay, France).

### 3.4. Total RNA Extraction, cDNA Synthesis and Quantitative Real-Time PCR

Frozen leaves and grape berry skins from control and treated grapevines were used to extract total RNA by following CTAB method [42]. Total RNA was cleaned up using RNeasy Mini Kit (Qiagen, Hilden, Germany) and DNase treatment to clean genomic DNA according to the instructions in the manual. Quality was checked on 1.0% agarose-gel to confirm the elimination of DNA traces, followed by NanoDrop 1000 Spectrophotometer quantification (NanoDrop Technologies, Inc., Wilmington, DE, USA) at A260/A280. Total of 1 µg DNase-treated RNA was used for cDNA synthesis by using Maxima First Strand cDNA Synthesis Kit. Gene specific primers were designed by QuantPrime program for expression analysis [43] (Table 1 and Supplementary Material 1). In addition, the qPCR primers were designed to span regions of exon-intron borders whenever possible, to ensure that no amplification of genomic DNA. Quantitative real-time PCR with 5x HOT FIREPol^®^ EvaGreen^®^ qPCR Mix Plus (ROX) (Solis Biodyne, Tartu, Estonia) was used to monitor the expression level of selected genes in triplicates using 3 µL 5× EvaGreen^®^ qPCR master mix. Each reaction contained 200 nm of each primer, 1 µL 10× diluted cDNA template and nuclease-free water to a final volume of 15 µL. Reactions were carried out on CFX96 Real-Time System C1000 Thermal Cycler (Bio-Rad, Irvine, CA, USA) with program: 95 °C for 12 min, 40 cycles of 95 °C for 15 s, 55 °C for 20 s and 72 °C for 20 s. The specificity of PCR reactions was checked through dissociation curves at the end of each qPCR reaction, by heating the amplicons from 65 to 95 °C. Gene expression was normalized to the Actin reference gene which has been recommended for data normalization in grapevine [44], using the ∆∆Cq method in CFX Manager Software 3.1 (Bio-Rad laboratories).

### 3.5. Statistical Analysis

JMP 11.0 (SAS Institute Inc, Cary, NC, USA) tool was used for statistical analysis in the present study, and two-way analysis of variance (ANOVA) was carried out for total polyphenols, anthocyanins and total tannin content in grape berries and leaves (*p* < 0.05). Gene expression data was subjected to independent t-test and significant differences were represented as asterisks on error bars. All the experiments were performed by using three replicates from the field (biological replicates) and a further three technical replicates from the lab, and expressed as mean ± standard deviation.

## 4. Conclusions

In conclusion, chitosan application in vineyards at veraison cessation stage stimulated the accumulation of phenolic compounds including anthocyanins in berry skins, by upregulating the expression of target genes in leaves and berry skins, involved in secondary metabolism and anthocyanin transport to the vacuoles. The exogenous application of chitosan may be used to induce the accumulation of secondary metabolites of oenological interest, for improved quality of grape berries and respective vine styles.

## Figures and Tables

**Table 1:** Annealing temperature, accession and primer sequences for the genes analyzed in present study.

Gene		Primer Sequence	Accession	Annealing Temperature
*Fe-SOD*	Superoxide dismutase	F 5′ CCTTTGTGAACCTAGGCGAACC 3′ R 5′ TGGCCGGGTTAGCTTGAACTC 3′	XM_019222444	55 °C
*Cu/Zn-SOD*	Superoxide dismutase	F 5′ AGATTGGCATGTGGTGTTGTTG 3′ R 5′ ACTCCCACATTACCCAACAACA 3′	NM_001281138.1	55 °C
*CAT*	Catalase	F 5′ GGTGTTCACACCTTCACTCT 3′ R 5′ GAGATCCTGAGTAGCATGACTG 3′	KP271927	55 °C
*APX*	Ascorbate peroxidase	F 5′ ATCTGGTGGTCATACTCTGG 3′ R 5′ TCTAGGAGAGCCTTGTCTGA 3′	XM_010655137	55 °C
*PAL*	Phenylalanine ammonia-lyase	F 5′ CCTACTGTTCAGAGCTCCAG 3′ R 5′ GCCACTAGGTATGTGGTAGACA 3′	XM_003633937	55 °C
*CHS*	Chalcone synthase	F 5′ CACTCTTCGAACTCGTCTCT 3′ R 5′ CCACCAAGCTCTTCTCTATG 3′	KT589834	55 °C
*F3H*	Flavanone3-hydroxylase	F5′CAGTGCAAGACTGGCGCGAGATCGTA3′ R 5′ TAGCCTCAGACAACACCTCCAGCAACT 3′	KY006128	52 °C
*ANR*	Anthocyanidin reductase	F 5′ CTGTCAGGTTCAGTCTCCAT 3′ R 5′ GTTGGGACTTTGTACTGAGG 3′	NM_001280956	55 °C
*UFGT*	UDP glucose: flavonoid 3-o-glucosyltransferase	F 5′ TGCAGGGCCTAACTCACTCT 3′ R 5′ GCAGTCGCCTTAGGTAGCAC 3′	GSVIVT0102441901	55 °C
*ABCC1*	Anthocyanin transporter	F 5′ CTCCACTGGTCCTCTGCTTC 3′ R 5′ AGCCTGCTTCGAAAGTACCA 3′	GSVIVT01028722001	55 °C
*MATE1*	Tonoplast transporter	F 5′ TGCTTTTGTGATTTTGTTAGAGG 3′ R 5′ CCCTTCCCCGATTGAGAGTA 3′	GSVIVT01028885001	55 °C
*GST*	glutathioneS-transferase	F 5′ AAGGATCCATGGTGATGAAGGTGTATGGC 3′ R 5′ AACTGCAGAAGCCAACCAACCAACAAAC 3′	GSVIVG01035256001	55 °C
*ACT*	Actin	F 5′ GTGCCTGCCATGTATGTTGCC 3′ R 5′ GCAAGGTCAAGACGAAGGATA 3′	GSVIVT010265800 01	55 °C

## Supplementary Materials

The following are available online at https://www.mdpi.com/1422-0067/21/1/306/s1, Figure S1: The bold lines are the melt curves for the amplicons and the thin lines are the melt curves for the primers (all are basal).
