# Supplementary material for: Chitosan Application in Vineyards (Vitis vinifera L. cv. Tinto Cão) Induces Accumulation of Anthocyanins and Other Phenolics in Berries, Mediated by Modifications in the Transcription of Secondary Metabolism Genes
Source: Int J Mol Sci. 2020 Jan 2;21(1):306. doi: 10.3390/ijms21010306 (PMC6981802; doi:10.3390/ijms21010306)
Supplement: Supplementary file 1 [file ijms-21-00306-s001.pdf]

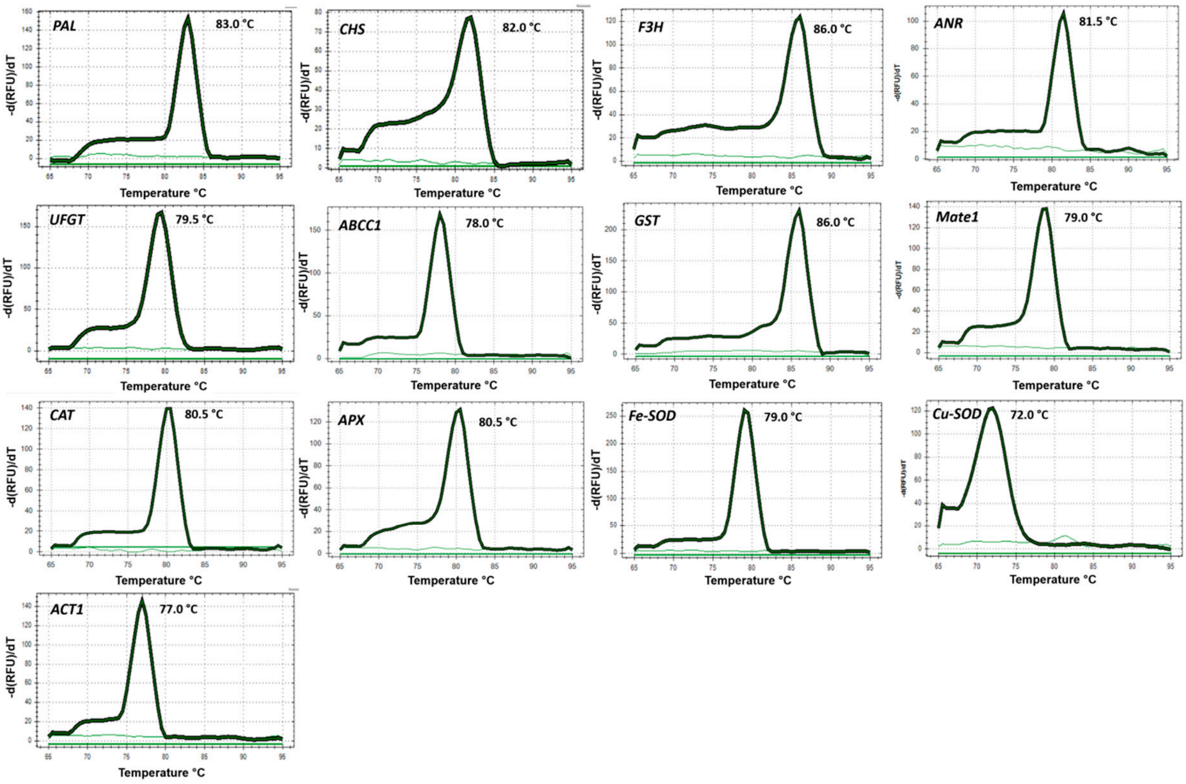

Figure 1. The bold lines are the melt curves for the amplicons and the thin lines are the melt curves for the primers (all are basal). Each primer represented the amplicon size, melting temperature and qPCR efficiency > 90%.
